# Author Correction: Tespa1 regulates T cell receptor-induced calcium signals by recruiting inositol 1,4,5-trisphosphate receptors

**DOI:** 10.1038/ncomms16183

**Published:** 2018-03-12

**Authors:** Jingjing Liang, Jun Lyu, Meng Zhao, Dan Li, Mingzhu Zheng, Yan Fang, Fangzhu Zhao, Jun Lou, Chuansheng Guo, Lie Wang, Di Wang, Wanli Liu, Linrong Lu

Nature Communications 8: Article number: 15732; DOI: 10.1038/ncomms15732 (2017); Published: 06 09 2017; Updated: 03 12 2018

In the original version of this Article, the affiliation details for Yan Fang were incorrectly given as “ZJU-UoE Institute, Zhejiang University School of Medicine, Hangzhou 310058, China”. This has now been corrected in both the PDF and HTML versions of the Article.

