# Supplementary material for: Tespa1 regulates T cell receptor-induced calcium signals by recruiting inositol 1,4,5-trisphosphate receptors
Source: Nat Commun. 2017 Jun 9;8:15732. doi: 10.1038/ncomms15732 (PMC5472764; doi:10.1038/ncomms15732)
Supplement: Supplementary Information — Supplementary Figures [file ncomms15732-s1.pdf]

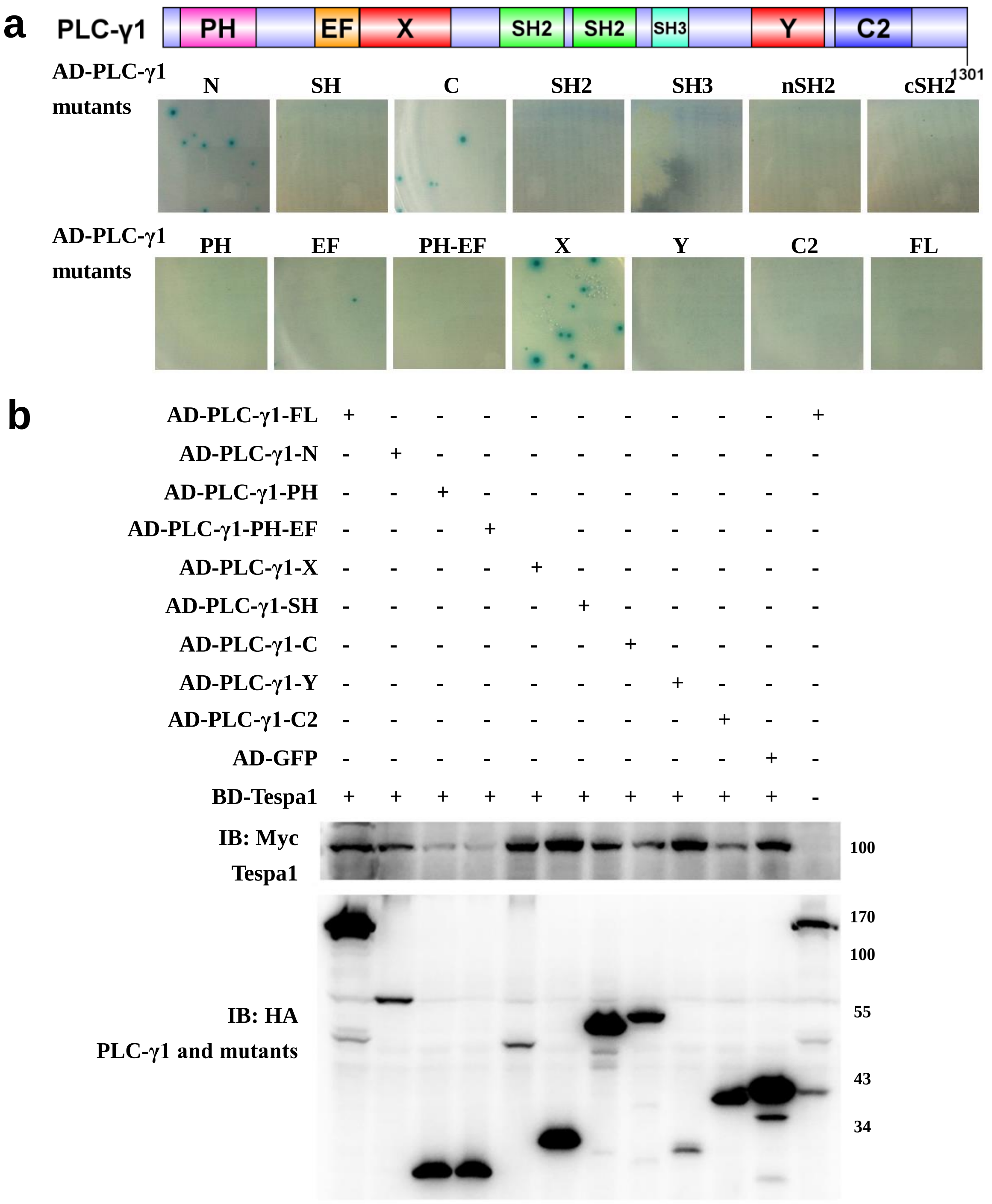

Supplementary Figure 1. Tespa1 interacts directly with PLC-γ1. (**a**) Interaction between pGBKT7-Tespa1 and pGADT7-PLC-γ1 or its different mutants in a two-hybrid system. The presence of clones on SD/-Trp-Leu-Ade-His/X-α-Gal/AbA (QDO/X/A) plates indicates an interaction between the bait and prey proteins. (**b**) Co-expression of pGBKT7-Tespa1 with pGADT7-PLC-γ1 or its different mutants in yeast, as detected by immunoblotting with anti-Myc and anti-HA antibodies. Data are representative of at least three experiments.

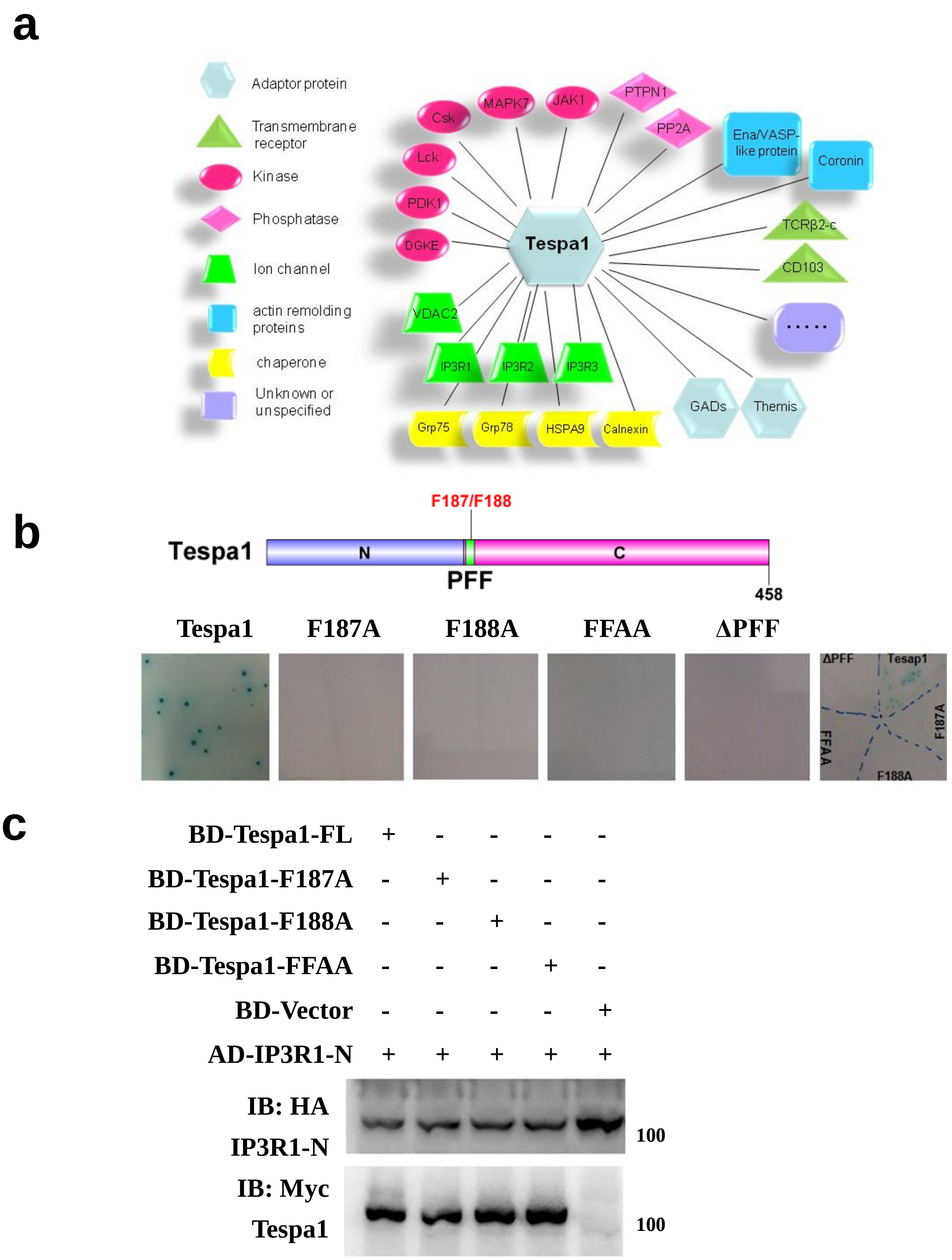

Supplementary Figure 2. Tespa1 interacts directly with IP3R1. (**a**) The network of Tespa1-interacting proteins isolated by immunoprecipitation with anti-Flag beads from Jurkat cells transfected with Flag-tagged Tespa1 and left unstimulated or stimulated for 5 min with anti-CD3 and anti-CD28. The immunoprecipitated proteins were analyzed by mass spectrometry, then classified according to function (left margin), such as adaptor protein, transmembrane receptor, tyrosine or serine-threonine protein kinase (Kinase), phospholipid or tyrosine phosphatase (Phosphatase), ion channel, actin remodeling protein, or chaperone. (**b**) Interaction between pGBKT7-Tespa1 or its different mutants and pGADT7-IP3R1-N (1-610) in a two-hybrid system. The presence of clones on SD/-Trp-Leu-Ade-His/X-α-Gal/AbA (QDO/X/A) plates indicates an interaction between the bait and prey proteins. (**c**) Co-expression of pGBKT7-Tespa1 or its different mutants with pGADT7-IP3R1-N (1-610) in yeast, as detected by immunoblotting with anti-Myc and anti-HA antibodies.

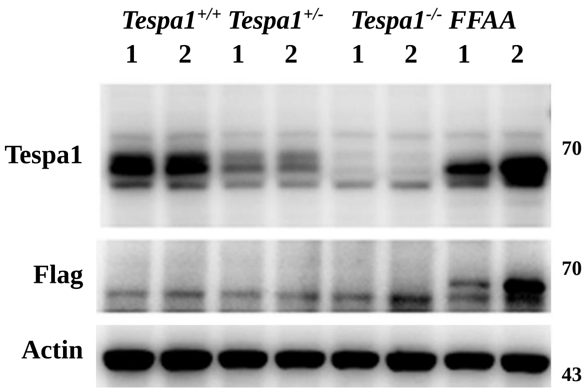

Supplementary Figure 3. Expression of the Tespa1-FFAA mutant in thymocytes. Immunoblotting was used to determine Tespa1 protein expression in sorted DP thymocytes from *Tespa1*$^{+/+}$, *Tespa1*$^{+/-}$, *Tespa1*$^{-/-}$, and *Tespa1-FFAA* mice expressing Flag-Tespa1-FFAA. 1 and 2 indicate two different mice.

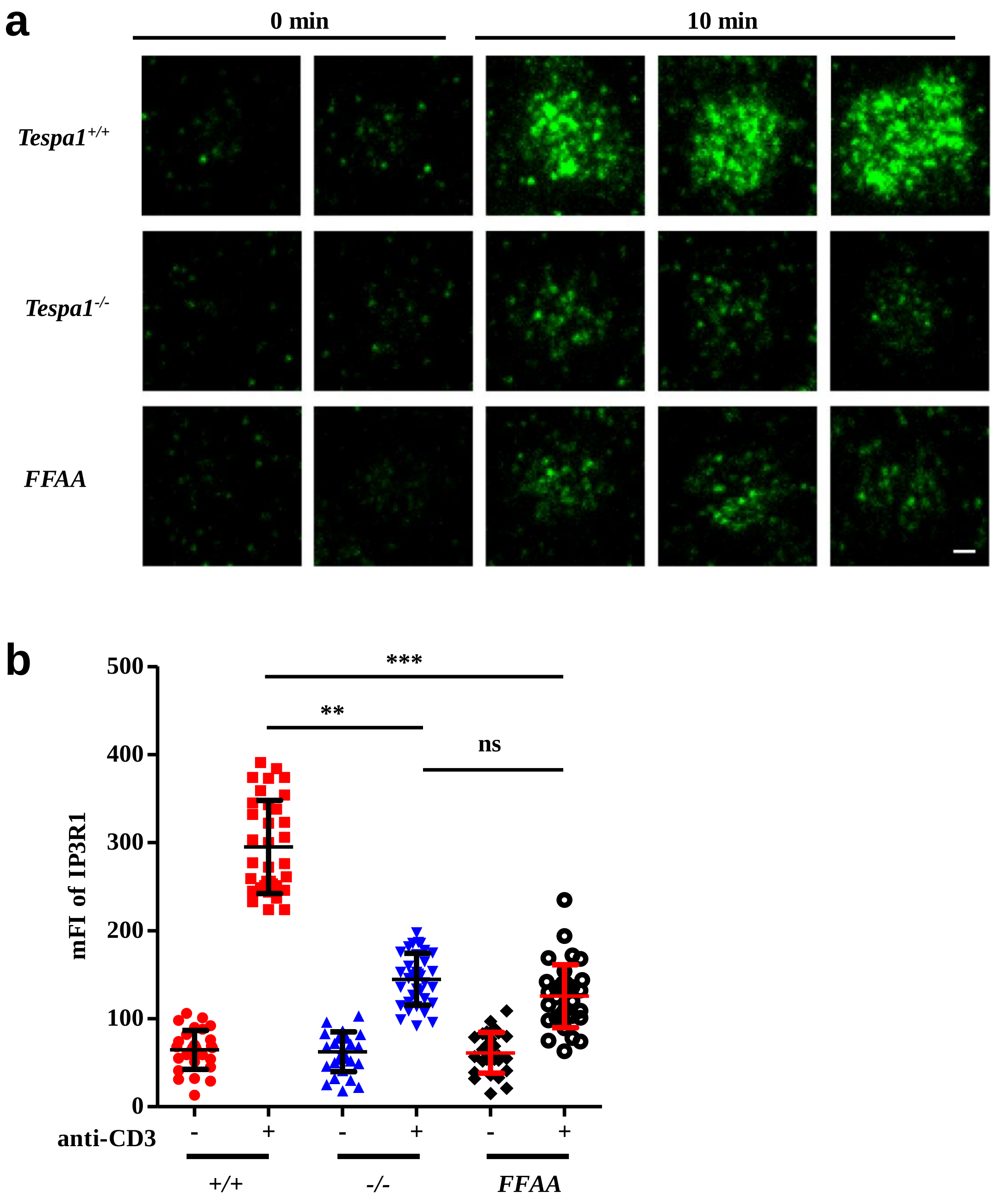

Supplementary Figure 4. Defective recruitment of IP3R1 to TCR-proximal region in *Tespa1*$^{-/-}$ and *Tespa1-FFAA* T cells. (**a**) Representative TIRFM images of IP3R1 (green) into the interface of T cells and the anti-CD3 coated coverslips in *Tespa1*$^{+/+}$, *Tespa1*$^{-/-}$ and *Tespa1-FFAA* T cells. Cells were activated for indicated times by anti-CD3-coated cover slips. Two (0 min) or three (10 min) representative cells are shown. Scale bar. 1.5 µm. (**b**) Mathematical quantifications for accumulation in mFI of IP3R1 microclusters are shown (n=34 for all stimulated groups from +/+, -/-, *FFAA*, n=30 for unstimulated group from +/+, -/- and n=28 for unstimulated group from *FFAA*). Three independent experiments with each dot represent the value of one cell. Small horizontal lines indicate mean ± s.d. One-way ANOVA was performed with a p value included.*p <0.05, **p < 0.01, ***p<0.001, ns, not significant.

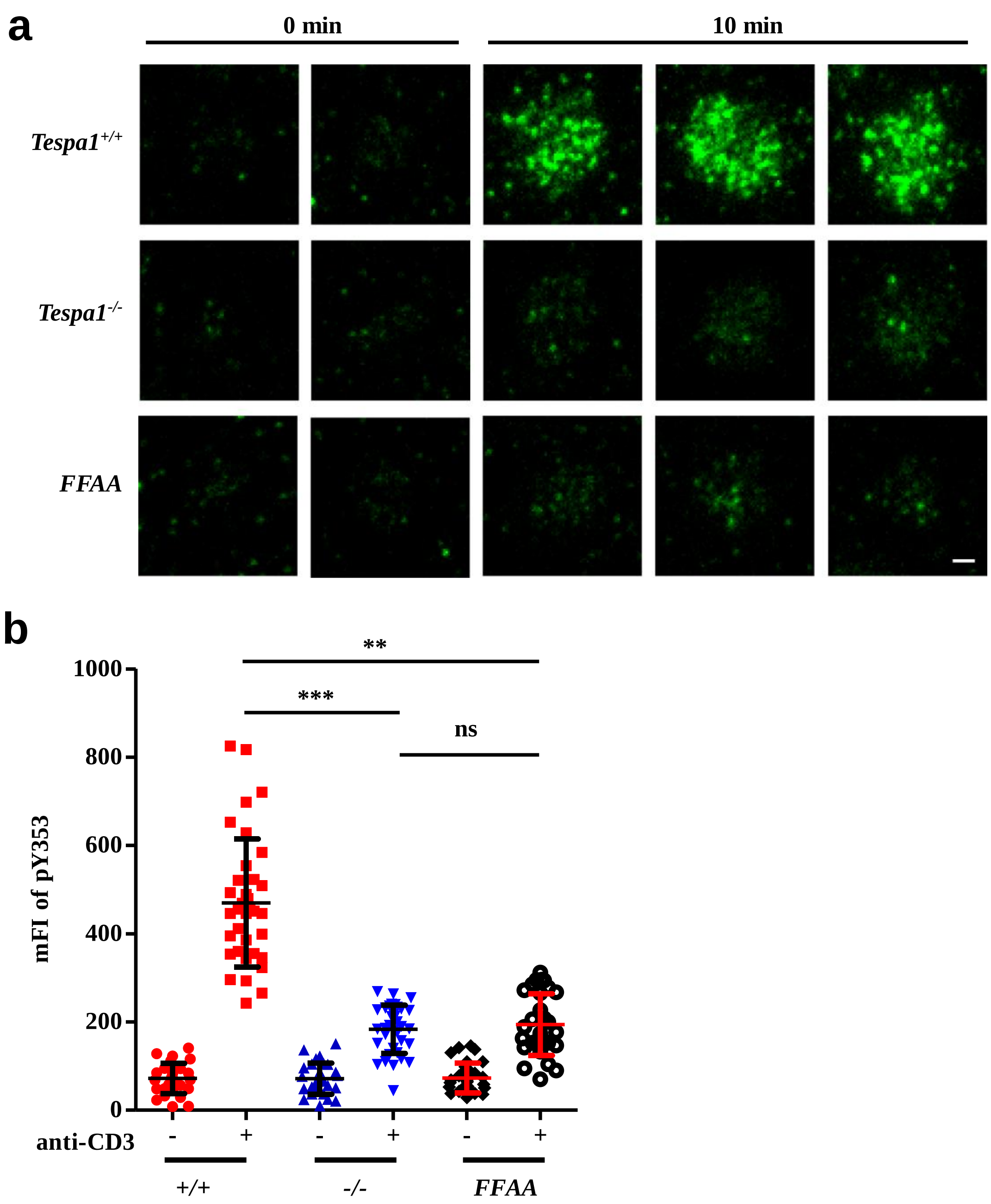

Supplementary Figure 5. Requirement of Tespa1 for Y353 phosphorylation of IP3R1. (**a**) Representative TIRFM images of IP3R1 pY353 (green) into the interface of T cells and the anti-CD3 coated coverslips in *Tespa1*$^{+/+}$, *Tespa1*$^{-/-}$ and *Tespa1-FFAA* T cells. Cells were activated for indicated times by anti-CD3-coated cover slips. Two (0 min) or three (10 min) representative cells are shown. Scale bar. 1.5 μm. (**b**) Mathematical quantifications for accumulation in mFI of pY353 microclusters are shown (n=30 for unstimulated groups from +/+, -/-, *FFAA* and stimulated group from *FFAA*, n=35 for stimulated groups from +/+, -/-). Three independent experiments with each dot represent the value of one cell. Small horizontal lines indicate mean ± s.d. One-way ANOVA was performed with a p value included.*p <0.05, **p < 0.01, ***p<0.001, ns, not significant.

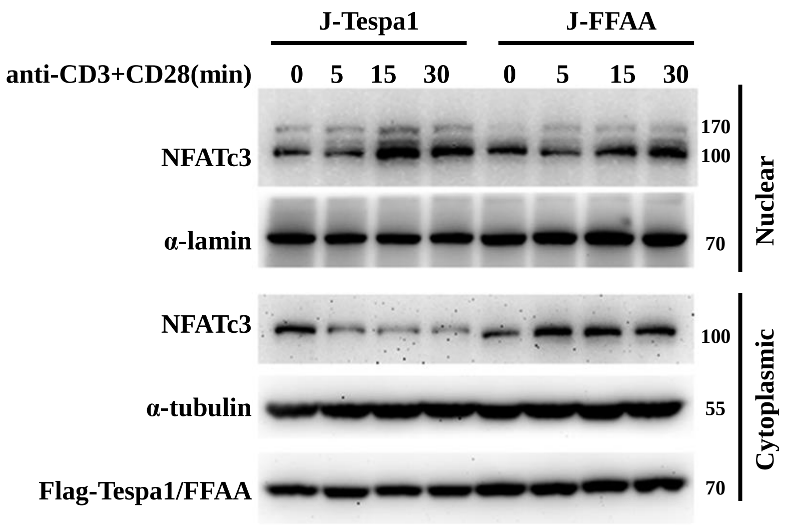

Supplementary Figure 6. Impaired translocation of NFAT1 from the cytoplasm to the nucleus in J-FFAA cells. Immunoblots (IB) of cytoplasmic and nuclear fractions of J-Tespa1, J-FFAA, and J-Vector cells left unstimulated or stimulated with anti-CD3 and anti-CD28 antibodies (each at 5 μg ml^{-1}) for the indicated times and probed with anti-NFATc3. Fractions were also immunoblotted with α-tubulin and α-lamin antibodies to confirm the purity of the cytoplasmic and nuclear fractions.

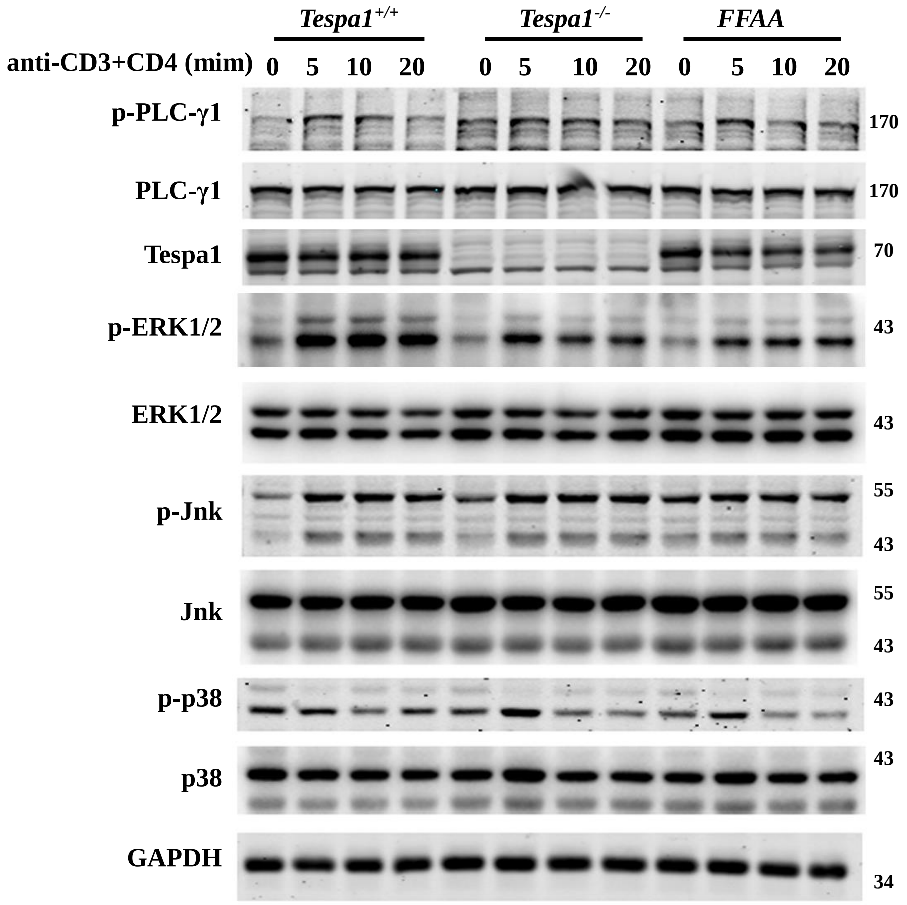

Supplementary Figure 7. Interruption of TCR-driven ERK activation in *Tespa1-FFAA* thymocytes. Immunoblots of p-PLC-γ1, p-ERK1/2, ERK1/2, p-p38, p38, p-JNK, and JNK in cell extracts from sorted *Tespa1*$^{+/+}$, *Tespa1*$^{-/-}$, and *Tespa1-FFAA* DP thymocytes left unstimulated (0) or stimulated for 5, 10, or 20 min with anti-CD3 and anti-CD4. Data are representative of >3 independent experiments.

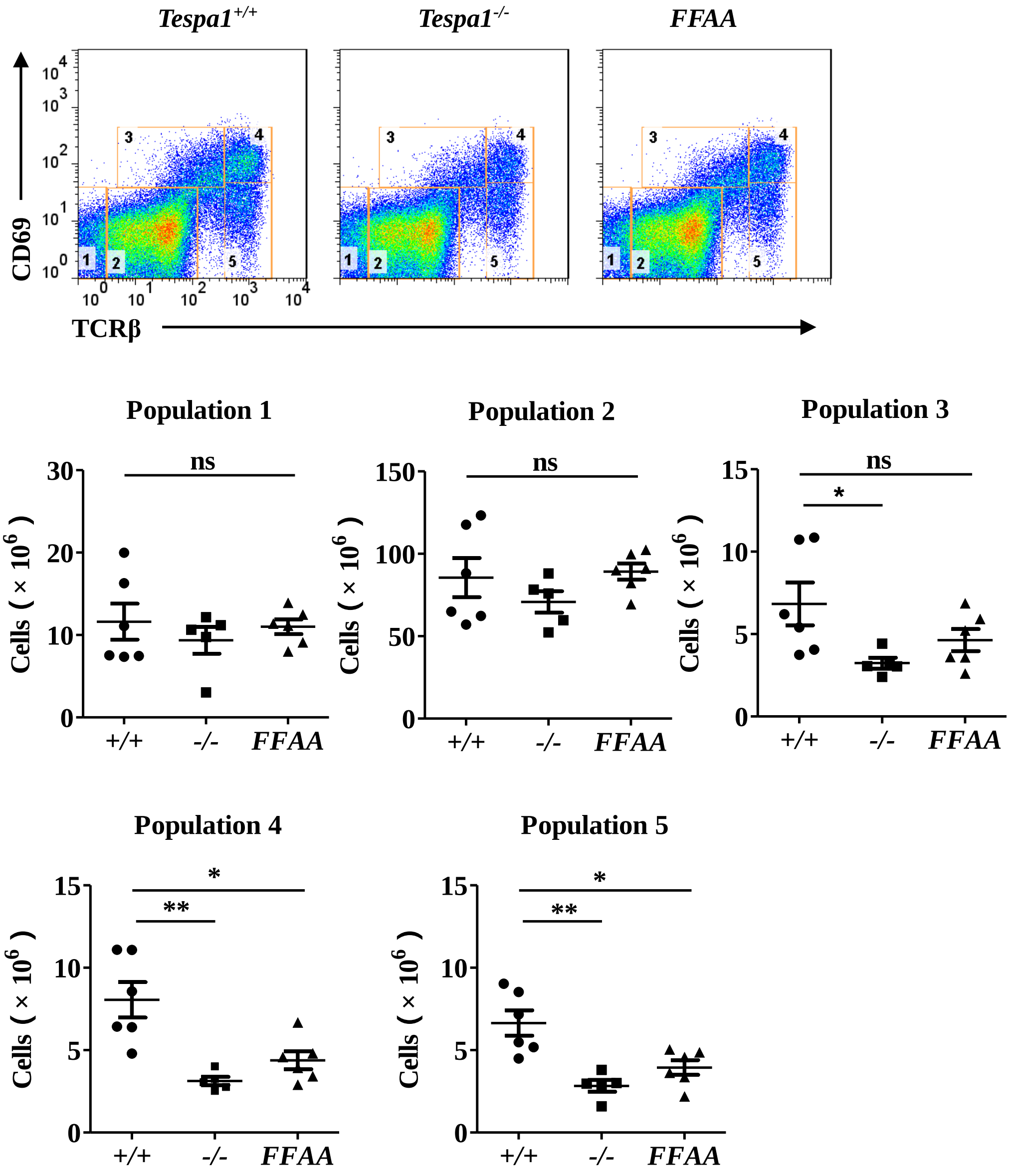

Supplementary Figure 8. Impaired positive selection in *Tespa1-FFAA* mice. Flow cytometric analysis of the surface expression of CD69 and TCRβ on *Tespa1*$^{+/+}$, *Tespa1*$^{-/-}$, and *Tespa1-FFAA* thymocytes; bottom, quantification of each subpopulation. *P < 0.05, **P < 0.01, ns, not significant (two-tailed t-test). Each symbol (a, c) represents an individual mouse; small horizontal lines indicate the mean (and s.d.).Data are representative of three independent experiments with 5-6 mice per group as the dots indicated

.

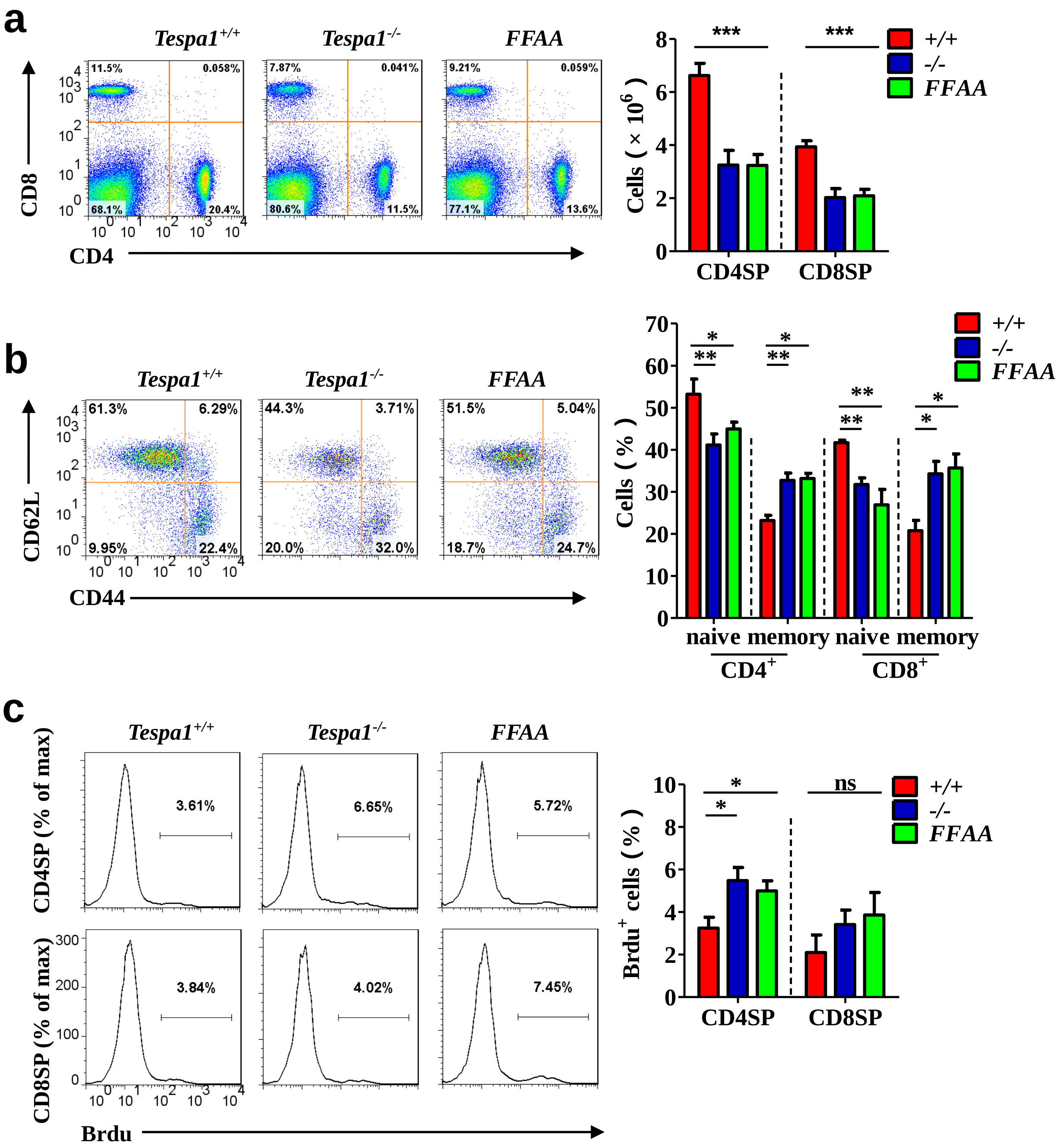

Supplementary Figure 9. Phenotype of peripheral T cells in *Tespa1-FFAA* mice. (**a**) Surface staining of CD4 and CD8 on *Tespa1*$^{+/+}$, *Tespa1*$^{-/-}$, and *Tespa1-FFAA* splenocytes (left) and percentages (middle) and total cell numbers (right, upper panel) of splenocyte subpopulations (n=7 for +/+, n=6 for -/- and n=8 for *FFAA*). (**b**) Expression of CD62L and CD44 on CD4^{+} splenocytes from *Tespa1*$^{+/+}$, *Tespa1*$^{-/-}$, and *Tespa1-FFAA* mice (left), and frequency of CD62L^{hi}CD44^{lo} and CD62L^{lo}CD44^{hi} subpopulations in the CD4^{+} and CD8^{+} populations (right). n=4 for +/+ and -/-, n=3 for *FFAA*. (**c**). BrdU uptake by CD4^{+} or CD8^{+} cells among *Tespa1*$^{+/+}$, *Tespa1*$^{-/-}$ and *Tespa1-FFAA* splenocytes (left). Right, frequency of BrdU^{+} CD4^{+} and CD8^{+} cells. *P <0.05, **P <0.01 and ***P <0.001 (two-tailed t-test). n=5 for +/+, *-/- and FFAA*. Data are representative of three independent experiments (error bars indicate s.d.)

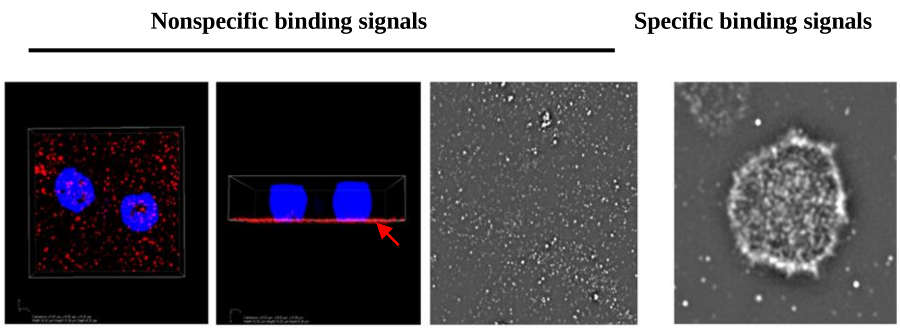

Supplementary Figure. 10. **3D display of the methods used to identify the focal plane.** Cells were treated and stained with different antibodies as described in the Methods. We set up a control group of cells stained only with secondary antibodies; nuclei were stained with DAPI (blue). The focal plane (the red arrow) was identified by nonspecific binding signals (red dots) between the secondary antibody and the coverslips (**left two pictures**). In this group, there were no specific signals for the primary antibodies in the focal plane (**the third picture**). In the experimental group, the specific signals for the primary antibodies were predominantly located in the focal plane and were captured (**the last picture**).

Fig 1b

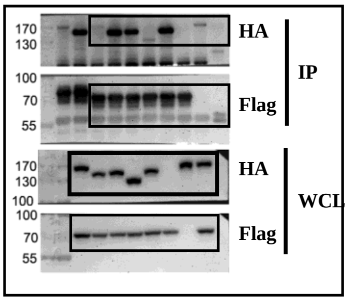

Fig 1c

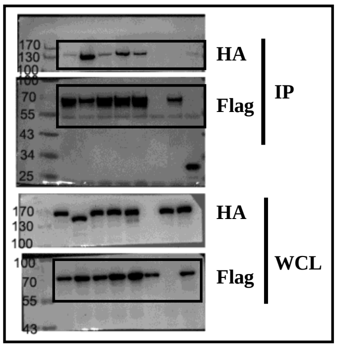

Fig 1d

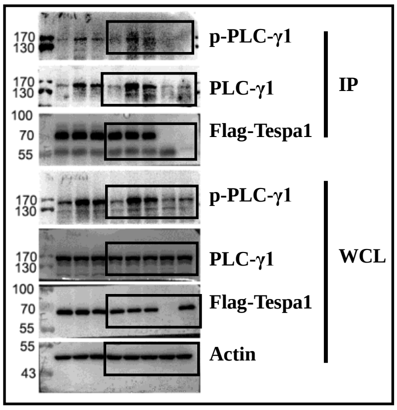

Figure 2a

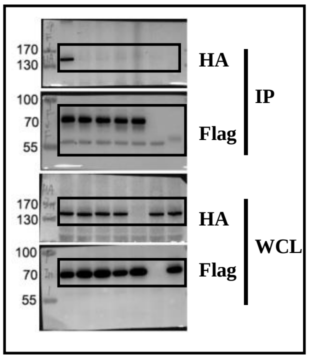

Figure 2b

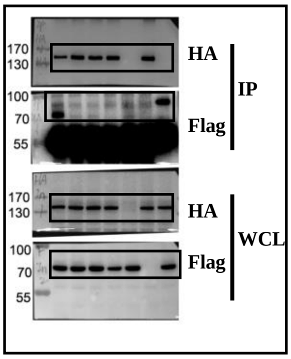

Figure 2c

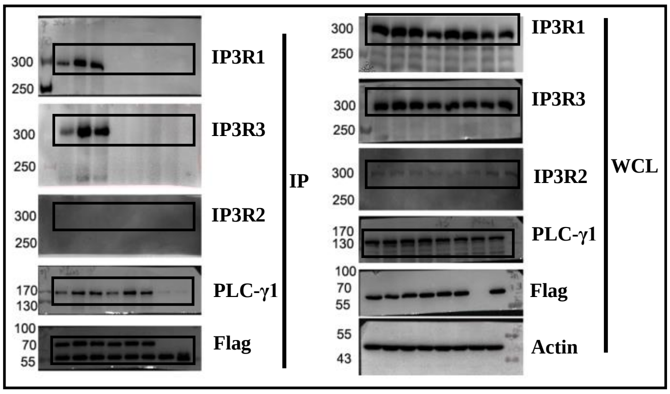

Fig 4d

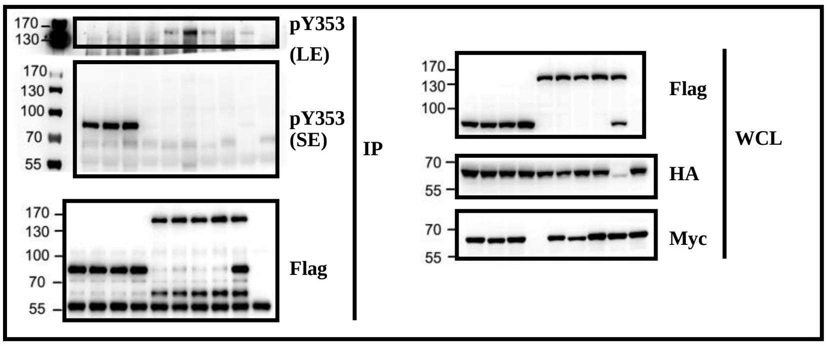

Supplementary Figure11. Uncropped scans of the western blots related to Figure 1, 2 and 4.

Supplementary Figure 1b

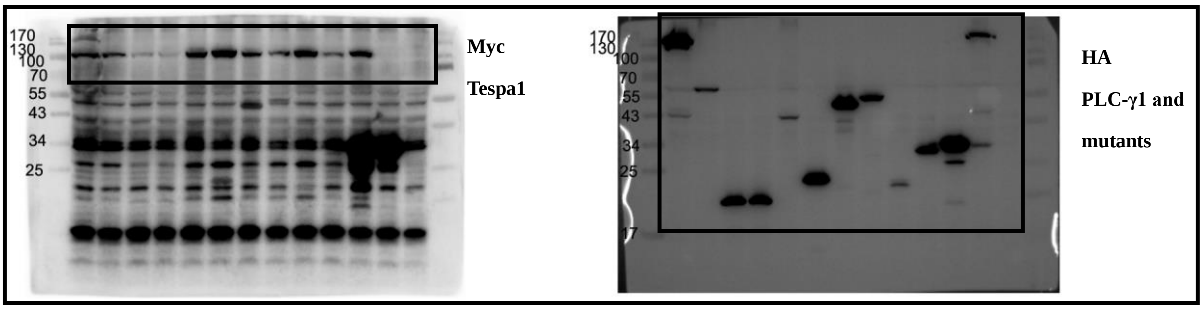

Supplementary Figure 2c

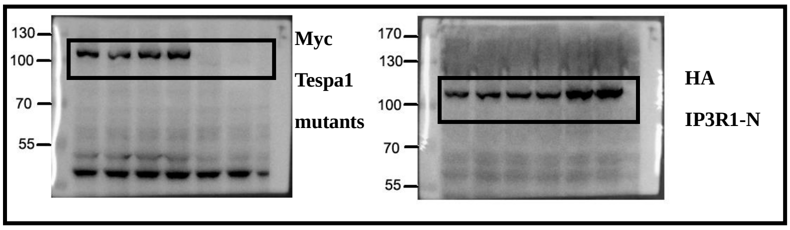

Supplementary Figure 3

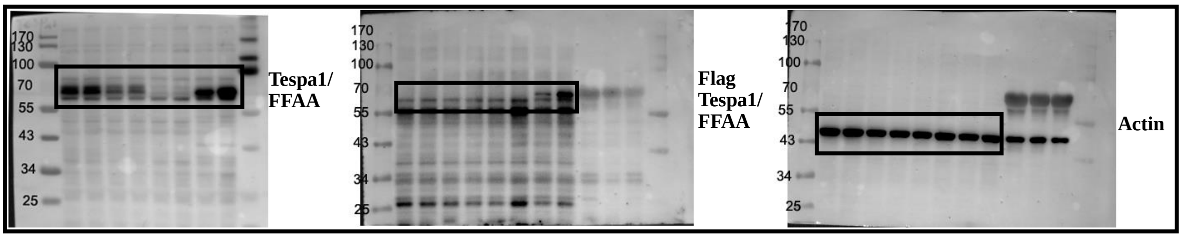

Supplementary Figure 6

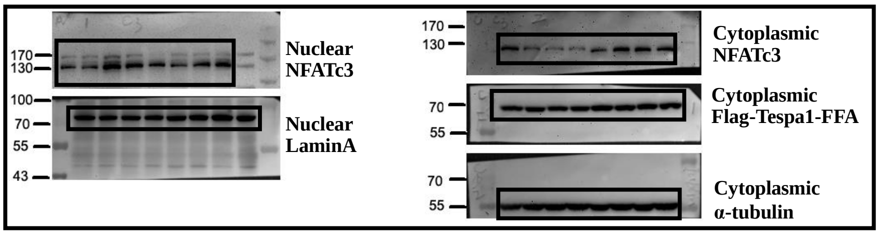

Supplementary Figure 7

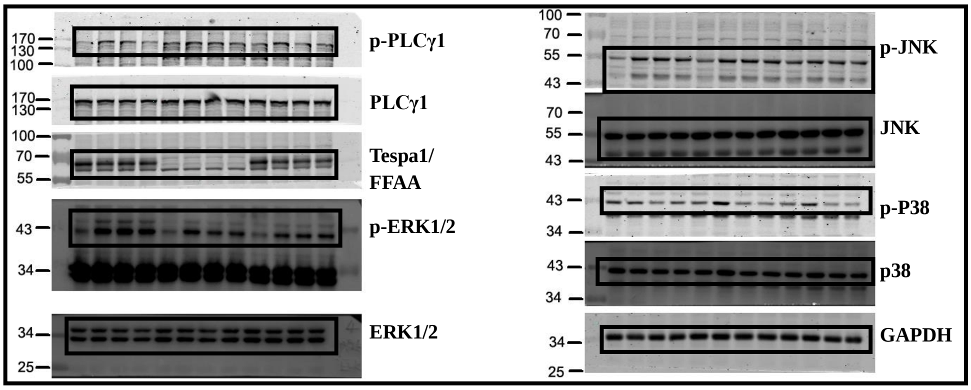

Supplementary Figure 12. Uncropped scans of the western blots related to Supplementary Figure 1-3, 6-7.
